# Expériences et perceptions de la mise à l’échelle d’une intervention de santé publique: entretiens auprès d’une variété d’acteurs français

**DOI:** 10.17269/s41997-025-01080-y

**Published:** 2026-04-29

**Authors:** Nolwenn Stevens, François Alla, Henri Bergeron

**Affiliations:** 1 Inserm U1218, BPH, Université de Bordeaux, CHU de Bordeaux, Bordeaux, France; 2 Centre de Sociologie Des Organisations, LIEPP, CNRS, Sciences Po, Paris, France

**Keywords:** Implementation science, Implementation research, Public health, Diffusion of innovation, Qualitative research, Science de la mise en œuvre, Recherche en implémentation, Santé publique, Diffusion des innovations, Recherche qualitative

## Abstract

**Introduction:**

Scaling up public health interventions is a major avenue for evidence-based public health policy. Many attempts have been made, but there have also been major difficulties and many failures. Scaling up is not easy; it is neither obvious nor spontaneous.

**Objectives:**

This study sets out to clarify the concept itself, distinguish the strategies on which it is based, describe the process, and identify the factors that affect it.

**Methods:**

Semi-structured interviews were conducted with 27 scale-up experimenters from a variety of backgrounds. The interviews focused on 19 public health interventions.

**Results:**

Scaling up is an active, progressive, and multidimensional process. It is based on a variety of strategies relating to territorial expansion, sustainability, and adaptation to the realities of the intervention. Four types of favourable decision could be identified: funding, support, commitment, and adoption; they emanate from multiple decision-makers. Eight essential activities emerged. In addition, scaling is based on organisations that adopt different conformations and different methods of identifying multipliers. Finally, six catalysts and six inhibitors were identified.

**Conclusion:**

This study has made it possible to stabilise the concept of scaling up and deepen our understanding of the process. New opportunities are emerging to support the players and decision-makers involved in scaling up public health interventions. Similarly, new research perspectives are emerging, in particular through the mobilisation of the political sociology of public action, and management and marketing sciences.

## Résumé

**Introduction:**

La mise à l’échelle des interventions de santé publique est une voie majeure des politiques de santé publique fondée sur les données probantes. De nombreuses tentatives sont identifiables, de même que de grandes difficultés et quantité d’échecs. La mise à l’échelle n’est pas aisée, elle n’est ni évidente ni spontanée.

**Objectifs:**

Cette étude s’attache à clarifier le concept lui-même, distinguer les stratégies sur lesquelles elle repose, décrire le processus et identifier les facteurs qui l’affectent.

**Méthodes:**

Des entretiens semi-directifs ont été conduits auprès de 27 expérimentateurs de la mise à l’échelle aux profils variés. Au cœur des récits, 19 interventions de santé publique.

**Résultats:**

La mise à l’échelle est un processus actif, progressif et multidimensionnel. Elle repose sur diverses stratégies portant sur l’expansion territoriale, la pérennisation et l’adéquation au réel de l’intervention. Quatre types de décision favorables ont pu être identifiées: le financement, le soutien, l’engagement et l’adoption; elles émanent de multiples décideurs. 8 activités essentielles sont apparues. Par ailleurs la mise à l’échelle s’appuie sur des organisations qui épousent différentes conformations et différentes méthodes d’identification des agents démultiplicateurs. Enfin 6 catalyseurs et 6 inhibiteurs ont été identifiés.

**Conclusion:**

Cette étude a permis de stabiliser le concept de mise à l’échelle et d’approfondir la compréhension du processus. De nouvelles opportunités émergent pour accompagner les acteurs et les décideurs impliqués dans la mise à l’échelle des interventions de santé publique. De même de nouvelles perspectives de recherche se dessinent notamment via la mobilisation de la sociologie politique de l’action publique, et les sciences de gestion et de marketing.

## Introduction

Voilà plus d’un demi-siècle que décideurs, chercheurs et acteurs poursuivent l’ambition de servir des politiques de santé fondées sur des données probantes, c’est-à-dire «*des décisions à grande échelle concernant les prestations offertes à la population et leur gestion*» (Banta, 2003; Oliver et al., 2014). Il en est de même dans le domaine de la santé publique (Banta, 2003). Cette stratégie est motivée par un devoir d’équité, de sécurité et d’efficacité et la nécessité de faire un usage raisonné des ressources. La mise en œuvre de politiques fondées sur des données probantes se confronte à de nombreuses difficultés qui sont richement documentées notamment concernant la mobilisation des données probantes dans les décisions (Oliver et al., 2014). A cette difficulté s’ajoute celle de la mise en application des décisions arrêtées. Il serait illusoire de penser qu’à la décision succède naturellement les faits et l’effet. Les politiques publiques reposent sur des décisions, définissent des objectifs à atteindre, s’adressent à des publics définis, s’inscrivent dans un cadre général d’action et sont constituées d’un ensemble de mesures concrètes qui en forme la substance (Mény & Thoenig, 1989; Muller, 2015). L’orientation arrêtée nécessite d’être opérationnalisée: c’est la question du choix des instruments d’action (Hassenteufel, 2011). Les interventions pour la santé des populations constituent des instruments importants pour l’opérationnalisation des politiques de santé publique. Ainsi la mise à l’échelle (MAE) des interventions constituerait la réalisation de cette stratégie politique. Toutefois, celle-ci n’est pas aisée. Il existe un véritable vivier d’expérimentations dont on ne sait précisément que faire (Stevens et al., 2021). Conservées dans leur état d’origine, ces interventions peuvent être exemplaires, leur impact demeure néanmoins anecdotique. C’est pourtant la visée de l’intervention en santé publique que d’être au bénéfice des populations (Hawe & Potvin, 2009). La MAE des interventions permettrait de concrétiser des progrès pour la santé des populations à partir d’expérimentations prometteuses (Milat et al., 2015; Stevens et al., 2022). Cela pose deux impératifs: être en capacité d’identifier les interventions d’intérêt, et être en capacité de mener à bien leur MAE. Or, aucun de ces impératifs n’est évident (Ben Charif et al., 2022; Milat et al., 2015). La MAE «ne va pas de soi», elle ne constitue pas l’évolution naturelle ou spontanée d’une intervention. C’est ainsi que de nombreuses interventions perdurent, parfois longuement, en tant qu’initiatives locales; d’autres interventions accèdent à la MAE sans avoir été pertinemment éprouvées; d’autres encore échouent lors de leur MAE malgré des résultats prometteurs lors de leur expérimentation (Dearing & Cox, 2018; Haut Conseil pour l’Avenir de l’Assurance Maladie., 2016; Ishimo, 2019; Mission interministérielle de lutte contre les drogues et les conduites addictives (MILDECA), 2019; Stevens et al., 2021; WHO & ExpandNet, n.d.-a). Le besoin de combler ces lacunes est prégnant (Ben Charif et al., 2022; Peters et al., 2013). Au cours des deux dernières décennies une littérature de plus en plus abondante est apparue sur la MAE des interventions de santé publique. Elle donne notamment à voir de riches travaux conceptuels, méthodologiques et pédagogiques. Cette littérature montre que la MAE est empruntée au service d’un triple enjeu: il est sanitaire (Aarons et al., 2017; Milat & Bauman, 2015; Milat et al., 2014, 2016, 2020; Yamey, 2011, 2012), éthique (Bradley et al., 2012; Hanson et al., 2003; Mangham & Hanson, 2010; Ovretveit et al., 2017; Paina & Peters, 2012) et économique (Aarons et al., 2017; Milat et al., 2020; Ovretveit et al., 2017). Prometteuse, la MAE a fait l’objet de divers travaux et productions. Certains concernent les démarches permettant d’établir la validité d’une intervention pour sa MAE au travers de la notion de potentiel de MAE ou «*scalability*» (Milat et al., 2013, 2020). D’autres sont centrés sur les démarches à entreprendre au cours de ce processus (Barker et al., 2015; Bradley et al., 2012; Cooley & Kohl, 2012; Indig et al., 2018; Milat et al., 2016; Nguyen et al., 2020; WHO & ExpandNet, n.d.-b). D’autres encore abordent les freins et leviers rencontrés dans cette aventure (Gericke et al., 2005; Hanson et al., 2003; Mangham & Hanson, 2010; Ovretveit et al., 2017; Yamey, 2011, 2012). Cette littérature est plus développée concernant les interventions mises en œuvre dans les pays à revenu faible ou moyen, et les MAE advenant dans une visée internationale. Ces travaux présentent la MAE comme un processus actif, qui s’opère via différentes voies: celles d’une MAE horizontale, verticale, organisationnelle ou fonctionnelle. La MAE est un processus non linéaire qui rassemble diverses phases de travail investies ou réinvesties en différents temps. Plusieurs freins et leviers sont mis en exergue, ils sont liés à l’intervention, au contexte, aux populations, aux ressources et aux stratégies de MAE.

En France, la volonté d’adopter une politique de santé publique fondée sur les données probantes a récemment été réaffirmée comme l’illustrent différentes initiatives telles que la mission de refondation de la santé publique en 2021,^1^ le dispositif article 51 de la loi de financement pour la sécurité sociale (LFSS) pour 2018,^2^ la révision actuelle du portail des interventions probantes en prévention et promotion de la santé porté par Santé Publique France depuis 2018^3^ ou la dynamique «Déploiement des interventions probantes et prometteuses en prévention et promotion de la santé» pilotée par la Société Française de Santé Publique depuis 2022^4^. Afin que la politique de santé publique fondée sur les données probantes ne reste pas une intention louable mais puisse être opérationnalisée, il est nécessaire d’explorer le concept ainsi que le processus de MAE et ses conditions. Quels enseignements pourront nous tirer des expériences de ceux qui œuvrent au quotidien à la MAE d’une ou plusieurs interventions dans le contexte français? De quelle façon ces témoignages résonnent ils avec la littérature existante sur le sujet?

## Objectifs

Les principaux objectifs de cette étude sont de i) préciser la notion de MAE; ii) distinguer différentes stratégies de MAE; iii) décrire le processus de MAE et iv) identifier les facteurs qui affectent le processus de MAE d’une intervention de santé publique.

Dans le cadre de cette étude, une intervention est définie comme «*un ensemble d'actions ayant un objectif cohérent pour provoquer un changement ou produire des résultats identifiables*» (Rychetnik et al., 2002). Nous ciblons les interventions de santé dites populationnelles. Elles correspondent à des interventions non cliniques qui s’intéressent aux programmes en ou hors secteur de santé avec un impact sur la santé des populations et non sur la santé des individus (Hawe & Potvin, 2009). Ces interventions sont généralement complexes telles que définies par le Medical Research Council. Cette complexité repose sur: i) le nombre de composantes en interaction, ii) l’influence des acteurs impliqués dans la mise en œuvre ainsi que de ceux qui la reçoivent, iii) le nombre et la variabilité des résultats attendus et iv) les adaptations pouvant survenir au cours de l’implantation des interventions (Craig et al., 2008).

Cette étude permet de compléter et dynamiser les résultats issus de travaux théoriques en interrogeant ceux qui, au quotidien, sont impliqués dans la MAE d’interventions de santé publique, et qui s’y investissent sans l’appui de cette littérature.

## Méthodes

Nous avons conduit une étude qualitative qui repose sur des entretiens conduits entre juillet et octobre 2023. Nous avons recueilli les récits d’expériences d’acteurs actuellement investis dans la MAE d’une intervention de santé publique, ou y ayant contribué par le passé. Les entretiens ont été conduit à distance et selon un mode semi-directif. Ils ont fait l’objet d’enregistrements puis de retranscriptions textuelles. L’ensemble des données a été rendu anonyme. Tous les interlocuteurs étaient volontaires pour participer à cette étude.

### Échantillonnage

Nous avons identifié nos interlocuteurs à partir d’interventions de santé publique ayant été repérées comme connaissant un processus de MAE via deux sources: le portail des interventions probantes et prometteuses de Santé Publique France et la liste des interventions promues dans le cadre du dispositif article 51 de la LFSS pour 2018. Nous avons ensuite sélectionné un échantillon d’interventions varié par les thématiques abordées, leurs populations cibles, les milieux de déploiement, leurs origines, ainsi que leurs stades et périmètres de déploiement. Pour chaque intervention, nous avons ensuite identifié des interlocuteurs dont l’expérience, l’expertise, le rôle passé ou actuel permettaient d’informer la MAE des interventions. De plus, nous avons eu le souci de diversifier les fonctions autour des interventions: concepteurs, opérateurs, évaluateurs, accompagnateurs, référents afin de multiplier les points de vue recueillis. Enfin l’échantillon construit initialement s’est enrichi au cours de la période de recueil, en invitant chacun de nos interlocuteurs à nous indiquer d’autres informateurs. Nous avons cessé le recueil en atteignant la saturation des données. Les acteurs identifiés ont été contactés par courriel entre juillet et septembre 2023 afin de leur proposer de participer à l’étude. Le contexte, les objectifs de l’étude ainsi que les modalités de participation ont été présentés dès la première prise de contact. Lorsque la première tentative de contact est laissée sans suite, une seconde tentative a été entreprise au cours du mois suivant. A l’issue de ces deux tentatives et sans réponse de la part de l’informateur sollicité, l’interlocuteur n’a plus été contacté et n’a donc pas intégré l’échantillon de l’étude.

### Outil de recueil

Le recueil a été structuré et systématisé par un guide d’entretien construit à partir d’un thème général décliné en sous-thèmes et étayés de questions de relance. Les échanges ont été structurés autour de trois grands axes: profil de l’interlocuteur, description de l’intervention et récit chronologique de la MAE. Ce guide repose sur les résultats d’une revue de littérature menée précédemment sur le concept. Tous les entretiens ont été menés par un même chercheur (NS).

### Analyse

L’analyse des entretiens a reposé sur une approche thématique structurée, choisie pour sa capacité à articuler une démarche théorique préexistante avec une ouverture à l’exploration inductive des discours. Ce choix méthodologique répondait à la visée de l’étude: comprendre en profondeur le phénomène de MAE à partir des récits d’acteurs, tout en s’appuyant sur un cadre conceptuel construit préalablement.

Les entretiens ont ainsi fait l’objet d’une analyse structurée à l’aide d’une grille évolutive, construite comme un cadre de codage. Cette grille permettait le report des verbatims essentiels selon les unités de sens déterminés à la fois a priori (issues du cadre théorique et du guide d’entretien) et *in itinere,* selon les éléments saillants du discours. Evolutive, cette grille d’analyse a été complétée et modifiée selon les propos recueillis permettant de rester au plus près de l’expérience des acteurs tout en assurant une cohérence dans l’analyse.. L’analyse des entretiens a été menée en deux temps. Une première analyse verticale de chaque entretien a été réalisée dans les jours suivants le recueil. Elle a permis une première structuration des thèmes récurrents ou marginaux et d’ajuster les techniques de recueil. Le second temps d’analyse réalisé après la collecte de l’ensemble du matériel, a permis une analyse horizontale des données. L’ensemble de l’analyse s’est inscrit dans une démarche articulant approche hypothético-déductive et approche inductive. La première par la construction de l’échantillon et des situations à explorer, ainsi que par la «*perspective ou sensibilité théorique*» construite au cours d’une revue de littérature menée précédemment (Anadon & Guillemette, 2007). La seconde par l’étude du phénomène de MAE à partir de l’expérience des acteurs, en donnant la priorité aux données recueillies (Anadon & Guillemette, 2007). Nous mobilisons donc une inférence abductive permettant «*de combiner de manière créative des faits empiriques avec des cadres heuristiques de référence*» et ainsi «*d’actualiser le travail créatif de la recherche qualitative tout en ayant recours aux connaissances existant dans le domaine auquel l’objet d’étude appartient*» (Brown & Kelle, 1997). Le codage des entretiens a été réalisée par une seule chercheuse (NS), garantissant l’unité de traitement du corpus. L’analyse horizontale a été discutée et enrichie par les trois chercheurs impliqués dans l’étude (HB, FA et NS), renforçant la validité interprétative des résultats.

## Résultats

Cette étude a permis de recueillir le témoignage de 27 interlocuteurs endossant une diversité importante de fonctions autour des interventions: concepteurs, opérateurs, chercheurs-évaluateurs ou référents. La plupart d’entre eux exercent au sein de structures associatives, certains travaillent pour l’agence nationale Santé Publique France, d’autres exercent en milieu de soins, d’autres encore évoluent dans le milieu de l’entreprise. Plusieurs d’entre eux ont changé de fonctions et de structures de rattachement au cours de la vie de l’intervention narrée. Ils sont également plusieurs à cumuler aujourd’hui différentes casquettes. Parmi les 27 expérimentateurs rencontrés, 24 jouaient actuellement un rôle dans la MAE de l’intervention au cœur du récit partagé et 3 n’avaient plus d’influence sur l’intervention. Le tableau 1 présente les missions auxquelles les expérimentateurs ont pris part, l’échelle de leurs actions et la temporalité de leur investissement dans le projet au moment de l’intervention. 22 entretiens ont été réalisés pour mener cette étude. Leur durée moyenne a été d’une heure quarante-cinq. Ces entretiens ont permis d’aborder 19 interventions de santé publique (tableau 2).

**Tableau 1:** Profilage des expérimentateurs

Expérimentateurs	Mission(s) MAE	Niveau d’action	Implication (au moment de l’entretien)
N°1	Formation à l’intervention	Départemental	Actuelle
N°2	Référent intervention Evaluation Dissémination	National	Passée
N°3	Référent intervention Conception Expérimentation	National	Actuelle
N°4	Référent intervention Conception Expérimentation	National	Actuelle
N°5	Référent intervention Expérimentation Formation Dissémination	National	Actuelle
N°6	Conception Référent intervention Evaluation Dissémination	National	Actuelle
N°7	Référent intervention Expérimentation Formation Dissémination Evaluation	National	Actuelle
N°8	Référent intervention Conception Expérimentation Dissémination	National	Passée
N°9	Référent intervention Dissémination	National	Actuelle
N°10	Expérimentation Formation Dissémination	Régional	Actuelle
N°11	Référent intervention Conception Expérimentation	National	Actuelle
N°12	Référent intervention Conception Expérimentation Dissémination	National	Actuelle
N°13	Référent intervention Formation Expérimentation Dissémination Evaluation	National	Actuelle
N°14	Référent intervention Conception Animation Expérimentation Formation Dissémination	Régional	Actuelle
N°15	Animation Formation Dissémination Evaluation	Régional	Passée
N°16	Référent intervention Formation Dissémination	Régional	Actuelle
N°17	Réfèrent intervention Dissémination Evaluation	National	Actuelle
N°18	Animation Référent intervention Formation Dissémination Evaluation	National	Actuelle
N°19	Dissémination	Régional	Actuelle
N°20	Conception Evaluation Référent intervention Dissémination	Régional	Actuelle
N°21	Référent intervention Formation Dissémination	National	Actuelle
N°22	Conception Référent intervention Formation Evaluation Dissémination	Régional	Actuelle
N°23	Référent intervention Dissémination	Départemental	Actuelle
N°24	Référent intervention Dissémination	Départemental	Actuelle
N°25	Référent intervention Conception Expérimentation Dissémination	National	Actuelle
N°26	Expérimentation	National	Actuelle
N°27	Expérimentation	National	Actuelle

**Tableau 2:** Description des interventions de santé publique analysées

Nom de l’intervention	Thème	Population	Milieu
I-CAPS (Intervention auprès des Collégiens centrée sur l'Activité Physique et la Sédentarité)	Activité physique	Enfants et jeunes de 3 à 18 ans	Collectivités, institutions, associations, établissements scolaires (scolaire, périscolaire extrascolaire)
PRIMAVERA	Addictions – conduites à risques et conduites addictives Développement des Compétences Psycho-Sociales (CPS)	Enfants et adolescents de 9 à 12 ans (CM1 à 5^ème^)	Scolaire
UNPLUGGED	Addictions – conduites addictives Développement des CPS	Collégiens de 6^ème^ et 5ème	Scolaire
LSPS (Lieux de Santé Promoteurs de Santé)	Promotion de la santé	Professionnels de santé et usagers des lieux de santé	Soins
LSST (Lieux de Santé Sans Tabac)	Addiction tabac	Professionnels de santé et usagers des lieux de santé	Soins
LIKE YOU	Image corporelle/alimentation –activité physique (nutrition)	Jeunes de 13 à 17 ans	Scolaire (cible) et périscolaire (occasionnellement)
NUTRITION PETITE ENFANCE	Nutrition	Professionnels de la petite enfance et parents	Garde petite enfance (crèches et assistantes maternelles)
GDVB (Grand Défi Vivez Bougez)	Activité physique	Adolescents	Scolaire
PANJO (Promotion de la santé et de l’Attachement des Nouveau-nés et de leurs Jeunes parents: un Outil de renforcement des services de PMI)	Parentalité – lien d’attachement	Professionnels de la Protection Maternelle et Infantile (PMI)/Familles (parents ou futurs parents)	Soins (PMI via visites à domicile)
SE&SR (Sortir Ensemble et Se Respecter)	Compétences psychosociales – violences dans les relations amoureuses	Jeunes de 13 à 18 ans	Scolaire, ainsi que dispositif d’apprentissage, mission locale etc.)
GBG (Good Behaviour Game)	Compétences psychosociales	Enfants en école élémentaire	Scolaire
PRODAS (PROgramme de Développement Affectif et Social)	Compétences psychosociales – développement affectif et social/ Santé mentale/Prévention et Promotion de la Santé	Enfants et adolescents	Scolaire (maternelle au lycée)
PSFP (Programme de Soutien aux Familles et à la Parentalité)	Bien être—santé mentale/pratiques parentales	Parents/enfants et adolescents (3–6 ans et 6-11ans)	Quartiers politiques de la ville prioritairement
COM’IN (COmpétences Mutuelles et INdividuelles)	Compétences psychosociales	Enfants et jeunes/acteurs sociaux et acteurs éducatifs	Territoire (quartier, bassin de vie etc.)
PM (Programme de lutte contre la Précarité Menstruelle)	Précarité menstruelle et santé sexuelle	Population vulnérable/acteurs de la veille sociale	Quartiers défavorisés/quartiers prioritaires
CECICS *(Cellule d’expertise et de coordination pour l’insuffisance cardiaque sévère)*	Parcours patients insuffisants cardiaques sévères – protocole de coopération	Patients insuffisants cardiaques	Soins
OCTAVE (Organisation Coordination Traitement Âgé Ville Établissements de Santé)	Parcours prise en charge médicamenteuse	Professionnels de santé/Sujets âgés	Soins
EQUILIBRES (EQUipes d'Infirmières LIBres REsponsables et Solidaires)	Paiement horaire des infirmiers dispensant des soins à domicile	Infirmiers	Soins
AS DU CŒUR	Activité physique adaptée/post rééducation cardiovasculaire	Personnes présentant un problème cardiovasculaire	Soins

### Le concept de mise à l’échelle

La MAE correspond à un processus actif, progressif, et multidimensionnel. La MAE est identifiée comme étant un processus actif qui nécessite des efforts délibérés et planifiés. Elle s’oppose donc à la représentation d’une évolution spontanée et passive de l’intervention. [Expérimentateurs 6,11,20] Les récits recueillis nous permettent d’identifier trois dimensions au processus de MAE: l’espace, le temps et le réel. Si la dimension territoriale de la MAE est systématiquement évoquée, ses frontières sont quant à elles très variables: du local à l’international. Elles correspondent aux aptitudes légales des porteurs ou des financeurs. La MAE poursuit un objectif de pérennité. Celle-ci est briguée par l’instauration d’un dispositif de soutien et d’accompagnement à la dissémination de l’intervention. Par ailleurs, la MAE reste fortement conditionnée par l’obtention de financements. [Expérimentateurs 5, 7, 11, 12,14, 16, 19, 21]. Dans la MAE, un souci particulier est porté au caractère réaliste de la proposition interventionnelle. Cela se traduit par une construction de l’intervention en adéquation avec les contraintes réelles qui influenceront son implantation. Cela se traduit également par de multiples tests: territoires différents et populations différentes. [Expérimentateurs 3, 4, 8, 9, 10, 13, 21] [Expérimentateurs 5, 10, 11] Enfin, la MAE s’inscrit dans une dynamique progressive: l’expansion territoriale de l’intervention est incrémentale; les changements de pratiques sont amenés graduellement et l’autonomisation des nouveaux opérateurs est croissante. [Expérimentateurs 7, 9,12,15,16,18,19,22].

### Les stratégies de mise à l’échelle

Les stratégies de MAE ont trois objets: l’expansion territoriale, la pérennisation et l’adéquation au réel.

Trois stratégies-types d’expansion territoriale ont été identifiées: l’opportunisme, le saupoudrage et la systématisation. Lorsque que la stratégie adoptée est celle de l’opportunisme, aucun objectif de dissémination n’est préalablement établi. La dissémination de l’intervention s’accomplit au gré des opportunités. [Expérimentateurs 5, 7, 13, 14] La stratégie de saupoudrage répond à l’objectif de disséminer l’intervention «un peu partout». L’objectif est moins tourné vers la quantité de territoires que sur la dispersion de l’intervention sur le territoire. [Expérimentateurs 1, 2, 5, 9, 16, 18, 21] Selon la stratégie de la systématisation, l’objectif poursuivi est celui d’atteindre 100% (ou le maximum) de la couverture cible. L’ambition est d’implanter l’intervention «partout». [Expérimentateurs 1, 2, 6, 9, 10] Le choix des stratégies adoptées correspond à des positionnements idéologiques différents. Schématiquement, certains prônent une conservation absolue de l’intervention d’autres son adaptation et intégration aux us courants. [Expérimentateurs 9, 14, 21] Le choix de l’une ou l’autre stratégie résulte également de contraintes pratiques liées aux ressources disponibles ou aux exigences des financeurs. [Expérimentateurs 9, 16].

Si la pérennisation de l’intervention ou la durabilité de ses effets sont des objectifs consensuels de la MAE, ils ne renvoient pas nécessairement aux mêmes procédés. On distingue deux orientations majeures. La première correspond à un processus d’autonomisation des opérateurs concernant la réalisation de l’intervention. [Expérimentateurs 1, 3, 5, 7, 9, 11, 19, 21, 22] La seconde poursuit un objectif d’acculturation et d’évolution plus diffuse des pratiques et des postures des opérateurs. Le pari implicite est que l’intervention s’efface au cours du temps tandis que les postures perdurent. Cette stratégie peut être investie d’emblée ou bien saisie dans un second temps. C’est alors bien l’intervention qui est transférée mais l’ambition est de modifier profondément les pratiques. Elle trace le sillon permettant de les normaliser. [Expérimentateurs 1, 2, 4, 7, 8, 13, 14, 15, 16, 17, 22].

Le processus de MAE repose également sur des stratégies d’adéquation au réel. L’une des exigences communément identifiées dans le processus de MAE est de garantir l’intégrité de l’intervention. Cet impératif impose aux acteurs démultiplicateurs, le respect du protocole interventionnel arrêté. Le degré de fidélité à l’intervention princeps est alors synonyme de qualité. [Expérimentateurs 4, 5, 7, 12, 15, 17, 18] Tout en prônant ce devoir de fidélité au modèle original, la nécessité d’adapter l’intervention pour son appropriation est soulignée. [Expérimentateurs 1, 2, 4, 5, 7, 8, 9, 10, 13, 14, 15, 16, 17, 19, 22] Cette double exigence questionne le socle identitaire de l’intervention. Dans ce processus, l’intervention est soumise à l’expérience du bateau de Thésée. La question du «squelette» de l’intervention apparait centrale lorsque l’on projette ou que l’on expérimente sa MAE. Pour certains l’emploi de termes tels que «réviser» ou «adapter» est devenu périlleux. D’autres, jonglant entre approche prescriptive et posture permissive laissent entrevoir l’idée de l’existence d’un seuil de tolérance, d’une quantité de modifications acceptable ou de types de modifications acceptables. Bien qu’une réflexion sur la substance active de l’intervention soit évoquée à plusieurs reprises, les méthodes employées à cette fin ne font pas l’objet d’une description précise. L’identification de ces «fonctions clés» résulte parfois d’un choix arbitraire. [Expérimentateurs 10, 13, 15, 17, 18] Les invariants relèvent tantôt des contenus de l’intervention, tantôt des modalités d’animation, des méthodes d’implantation ou des principes et objectifs interventionnels. [Expérimentateurs 4, 5, 7, 8, 10, 14, 15, 16, 17, 18] Face à ces différents constats et difficultés, la pertinence du ciblage de l’unité «intervention» comme objet de la MAE est parfois contestée. Cette remise en question se fait à la faveur d’une approche plus globale, embrassant différentes interventions comme autant de possibilités d’agir face à un problème de santé publique. Elle se fait également à la faveur d’un déport de la systématisation vers la phase du diagnostic de santé publique ou d’analyse des besoins et de la mise à disposition d’acteurs compétents pour y répondre. Elle se déplace sur la formation portant sur les compétences et les postures professionnelles pertinentes. Dans ces cas, l’évaluation de l’efficacité des activités déployées se pense en termes d’impact populationnel avant tout. [Expérimentateurs 1, 2, 8, 9, 14].

### Le processus de mise à l’échelle

Le processus de MAE suppose le concours de différentes opérations: des décisions, des organisations et des activités (tableau 3).

**Tableau 3:** Activités au cœur de la mise à l’échelle des interventions de santé publique

Communication	Des activités de communication sont menées envers différentes cibles: bénéficiaires, acteurs démultiplicateurs (responsables des sites de déploiement, opérateurs, structures ou agents relais), décideurs, financeurs ou institutions. Elles ont pour but la promotion de l’intervention	[Expérimentateurs 1, 3, 4, 5, 6, 7, 9, 10, 11, 12, 13, 14, 15, 16, 17, 19, 20, 21, 22]
Coordination	La MAE repose sur d’importantes missions de coordination de l’échelon le plus local et opérationnel à l’échelon le plus central et stratégique. Il s’agit d’organiser les ressources nécessaires à la MAE, d’orchestrer les activités et de synchroniser les différents acteurs	[Expérimentateurs 1, 2, 3, 4, 6, 9, 10, 11, 13, 16, 19, 20, 21, 22]
Contractualisation	La MAE implique souvent l’établissement de multiples contrats ou conventions. Il s’agit de régir l’octroi des financements, la propriété intellectuelle et le respect de l’intégrité de l’intervention, les partenariats ou les prestations. Tous ont pour objectif de réguler les agissements des différentes parties impliquées dans la MAE de l’intervention en fixant les droits et les devoirs de chacun	[Expérimentateurs 1, 2, 10, 14, 16, 17, 19, 21]
Formation	La formation est également centrale. Les dispositifs abordent différents aspects: contenus thématiques et apports théoriques; techniques d’animation, gestes et postures professionnelles; méthodes et conditions d’implantation et stratégies pédagogiques	[Expérimentateurs 1, 2, 4, 5, 7, 10, 11, 12, 13, 14, 15, 16, 17, 19, 21, 22]
Outillage	La MAE d’une intervention s’appuie sur de nombreux outils. Ils facilitent l’adoption et la dissémination de l’intervention et contribuent également à l’harmonisation des pratiques	[Expérimentateurs 1, 2, 3, 5, 6, 7, 9, 10, 11, 12, 13, 14, 15, 16, 17, 19, 20, 21]
Actualisation	Le processus de MAE requière une veille constante sur l’état des connaissances et des pratiques, afin de mettre en œuvre les révisions requises. Ainsi la MAE suppose l’actualisation continue de la proposition interventionnelle et de l’ensemble des productions afférentes: formations, techniques et outils	[Expérimentateurs 1, 5, 9, 10, 12, 14, 17, 19, 21]
Facilitation	La structure référente prodigue un accompagnement aux opérateurs afin de sécuriser leur pratique, de leur permettre d’acquérir de nouvelles compétences et de devenir autonomes. Ces activités peuvent prendre diverses formes: conseils, mise en place d’un contact d’assistance type «hotline», mentorat ou séances d’analyse de pratiques par exemple	[Expérimentateurs 1, 2, 3, 4, 5, 7, 10, 11, 12, 13, 14, 15, 16, 17, 18, 19, 20, 21, 22]
Monitorage	Le monitorage de la MAE se traduit par une surveillance continue tant de l’implantation de l’intervention que de sa dissémination et de sa pérennisation. Ce suivi peut être qualitatif, il permet alors d’observer la mise en œuvre de l’intervention et d’identifier les difficultés et les besoins des nouveaux opérateurs. Il peut également être quantitatif permettant alors de colliger des données d’activité: public touché, nombre d’opérateurs ou de sites actifs, accompagnements assurés, formations dispensées mais aussi l’envergure de la dissémination et sa pérennité	[Expérimentateurs 1, 5, 6, 7, 9, 10, 11, 13, 14, 16, 19, 21, 22]

Les décideurs de la MAE sont nombreux: responsables politiques, institutions de santé publique, opérateurs et parfois bénéficiaires; et les décisions revêtent différentes formes. La MAE mobilise un patchwork de financements et de financeurs variés. Les fonds octroyés peuvent bénéficier à différents protagonistes. [Expérimentateurs 1, 2, 3, 5, 6, 7, 9, 10, 11, 13, 14, 15, 16, 17, 19, 20, 21, 22]. La décision peut prendre la forme d’un soutien, ou d’une validation de l’intervention et de sa MAE, émanant d’autorités politiques, d’agences, de chercheurs ou d’associations du domaine. Ce type de décision nourrit la légitimité de la MAE de l’intervention. [Expérimentateurs 1, 2, 3, 5, 7, 9, 10, 11, 13, 14, 17, 19, 20] Rares sont les récits qui font mention de décisions engageantes par lesquelles le décideur prend la responsabilité de la MAE. La décision astreignante est parfois mentionnée comme une espérance. Certains expérimentateurs expriment le regret que les autorités politiques n’imposent pas davantage l’adoption de certaines interventions comme c’est le cas par exemple pour le programme pHARe sur le harcèlement qui est devenu une obligation pour tous les établissements scolaires. [Expérimentateurs 1, 3, 4, 6]. Une autre forme de concrétisation de la décision, est celle d’adopter l’intervention. La MAE d’une intervention n’étant pas—dans la majorité des cas—régit par la contrainte mais par la libre adoption des opérateurs, celle-ci est déterminante. [Expérimentateurs 1, 4, 5, 6, 7, 9, 10, 11, 12, 14, 16, 17, 19, 20, 21].

On distingue plusieurs méthodes d’identification des acteurs démultiplicateurs: la désignation, la sélection et l’adoption libre. Elles participent d’un continuum établi entre une approche dite «*top down*», pour la désignation, c’est-à-dire où l’impulsion dominante est du côté des institutions et l’approche «*bottom up*» concernant l’adoption libre où l’impulsion dominante vient des acteurs de terrain. La méthode dite de sélection est à la croisée de ces deux impulsions. Enfin le démarchage est davantage un déclencheur du ralliement à l’intervention. La désignation peut concerner les structures supports, les structures relais, les opérateurs ou les sites d’intervention. [Expérimentateurs 1, 9, 13, 15, 16, 18, 21, 22] La sélection résulte de l’étude de candidatures pouvant être issues d’appels à projets ou d’appels à manifestation d’intérêt. [Expérimentateurs 2, 9, 13, 18, 21] L’adoption libre repose sur le volontariat. [Expérimentateurs 1, 2, 4, 5, 12, 14, 15, 21, 22] Cette méthode profite d’une transmission informelle dite de «bouche-à-oreille». [Expérimentateurs 5, 7, 9, 16, 20, 22] Enfin, la dernière méthode identifiée est celle du démarchage selon laquelle la structure support adopte une posture pro-active et invite les acteurs et les sites potentiels de déploiement à mettre en œuvre l’intervention. [Expérimentateurs 6, 7, 8, 9, 11, 12, 15, 16, 19, 20, 22] Par «acteurs démultiplicateurs» nous désignons l’ensemble des professionnels qui joue un rôle actif dans la MAE d’une intervention. Ces acteurs démultiplicateurs peuvent toutefois assumer des missions différentes, certains agissent sur le terrain par l’animation de l’intervention auprès des bénéficiaires, d’autres endossent un rôle de pilotage de la MAE agissant ainsi de façon plus distale.

Différentes conformations organisationnelles peuvent être empruntée permettent d’organiser l’opération de MAE, d’identifier clairement la distribution et l’articulation des missions entre chaque acteur. Ainsi, par «conformation» nous désignons l’ensemble des acteurs du système de MAE d’une intervention, la répartition et l’articulation de leurs activités. On distingue une conformation «pyramidale» et une conformation «rayonnante», plus rarement empruntée. Dans ces deux conformations-types un souci de proximité est avancé. La première y répond en piochant les différents acteurs de la MAE au sein du territoire concerné, et la seconde par un mouvement «d’aller vers». Dans les deux conformations on identifie une structure support pour la MAE qui est référente de l’intervention et pilote la dissémination.

**Fig. 1:**
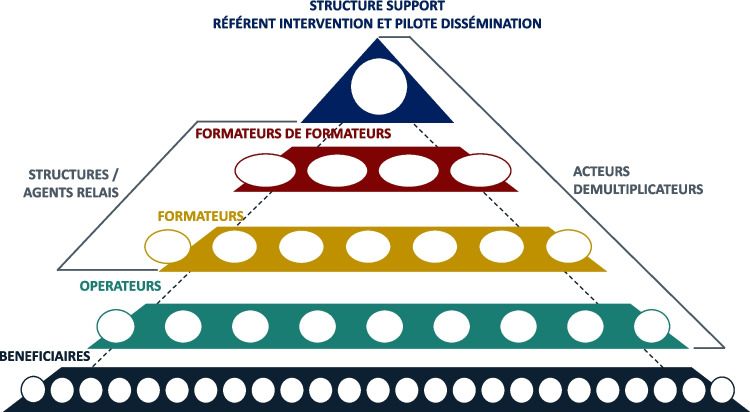
Conformation pyramidale pour la dissémination d’une intervention

Selon la conformation pyramidale (Fig. 1), à chaque strate d’acteurs correspond des missions et un niveau territorial. Plus l’acteur se positionne à proximité du sommet, plus l’étendue de sa zone de responsabilité est grande. Plus l’acteur se positionne à proximité de la base, plus ses interactions avec le terrain sont importantes. [Expérimentateurs 1, 5, 6, 7, 12, 15, 17, 19, 21].

**Fig. 2:**
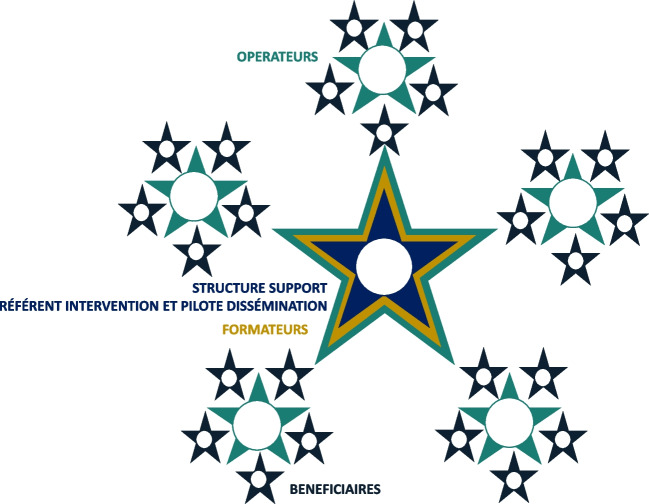
Conformation rayonnante pour la dissémination d’une intervention

Selon la conformation rayonnante (Fig. 2) la structure support est davantage impliquée sur le terrain. Elle est elle-même formatrice voire opératrice et se déplace sur les différents sites de déploiement. Elle joue un rôle de colporteur de l’intervention. [Expérimentateurs 2, 8, 17, 18, 20].

Enfin, on compte huit activités au cœur de la MAE: la communication, la coordination, la contractualisation, la formation, l’outillage, l’actualisation, la facilitation, et le monitorage (tableau 3).

### L’évaluation

La nécessité d’évaluer l’intervention et sa MAE fait consensus, pourtant les évaluations sont rarement effectives. Les motifs invoqués sont un manque de financements, de temps ou de savoir-faire. L’évaluation peut porter sur l’intervention, son implantation ou sa dissémination. Les témoignages recueillis ne font pas mention d’évaluation concernant la pérennisation. Lorsque l’intervention est l’objet de l’évaluation, l’objectif est de valider le «prototype» interventionnel qui sera porté à plus grande échelle. [Expérimentateurs 1, 2, 3, 4, 5, 6, 7, 8, 9, 10, 11, 12, 13, 14, 15, 17, 18, 21, 22]. Concernant son implantation, l’objectif est de définir les stratégies de mise en œuvre de l’intervention. [Expérimentateurs 2, 3, 7, 9, 10, 14, 15, 17, 19, 20, 21] Le troisième objet plus rarement saisi, à regret souvent, est la dissémination de l’intervention ainsi que le dispositif la permettant. Les objectifs sont d’apprécier l’évolution de la couverture de l’intervention et d’analyser les stratégies et méthodes employées à cette fin. [Expérimentateurs 1, 7, 9, 13, 17, 20, 21].

Par ailleurs, les méthodes évaluatives adoptées posent question. Dans de très nombreux cas, l’intervention et sa MAE sont éprouvées dans le cadre de dispositifs expérimentaux. Le caractère dérogatoire de ces dispositifs représente une opportunité pour déployer des interventions audacieuses. Parce que les tests interventionnels supposent des engagements moindres, ces formats sont plus facilement saisis. Ainsi on retrouve sous couvert d’expérimentations, des déploiements assez massifs. Le caractère dérogatoire de l’expérimentation permet également de remettre en question l’existant et de déplacer les cadres en vigueur. [Expérimentateurs 3, 12, 17] D’un autre côté, le caractère dérogatoire des dispositifs expérimentaux brouille la validité externe des résultats. L’expérimentation régit plusieurs aspects majeurs de l’intervention et de sa MAE tels que: la sélection des sites de mises en œuvre et des futurs opérateurs et l’application d’un protocole d’intervention. Le contrôle de ces paramètres concoure à la réunion de conditions favorables qui ne correspondent pas cependant au «monde réel». La dynamique expérimentale influence également l’adhésion des parties prenantes. Dès lors se pose la question de ce qui est réellement observé et évalué. [Expérimentateurs 2, 3, 4, 8, 10, 13, 15, 17].

### Les catalyseurs de la mise à l’échelle

La MAE est favorisée par un ensemble de conditions liées à l’intervention, aux protagonistes intervenant au cours du processus et au contexte.

#### Une histoire de confiance

Le processus de MAE est facilité lorsque l’intervention apporte des gages de confiance. Trois éléments y contribuent: la qualification d’intervention «probante», des résultats positifs concernant l’impact de l’intervention, et enfin le respect de «bonnes pratiques» de santé publique. Décrocher le titre d’intervention «probante» apparait comme un sésame pour la MAE, encourageant l’adoption comme le financement de l’intervention. Ainsi une véritable quête à l’estampille «probante» s’amorce avec le projet de MAE. Cependant, la procédure par laquelle le titre de «probant» est attribué est mal identifiée, de même que les caractéristiques concourant à cette dénomination. Ainsi, c’est moins le souci de colliger les conditions à l’obtention de l’étiquette qui occupe les esprits que celui de recevoir le titre lui-même. [Expérimentateurs 1, 5, 8, 14, 16, 17, 18, 21] Par ailleurs, pour que l’intervention gagne la confiance des différents protagonistes, elle doit avoir été éprouvée par de solides dispositifs d’évaluation. Les principales questions évaluatives explorées ont trait aux résultats de l’intervention. Si l’identité des évaluateurs joue un rôle important dans le crédit accordé à l’intervention, les designs d’étude épousés ne font l’objet d’aucune analyse critique. [Expérimentateurs 2, 3, 6, 8, 9, 12, 13, 15, 16, 17, 21] Le dernier élément permettant d’alimenter la confiance en l’intervention est le respect d’un ensemble de bonnes pratiques (principes éthiques et interventionnels) de santé publique. [Expérimentateurs 3, 12, 13, 14, 15, 22].

#### Du changement

Le caractère novateur de l’intervention peut être un atout pour sa MAE, en séduisant les futurs opérateurs plus enclins à adopter une intervention originale, capable de bousculer leur routine et de dynamiser leur pratique. [Expérimentateurs 3, 9, 10, 11, 14, 21, 22].

#### Une question de réalisme

La mesure dans laquelle la mise en œuvre de l’intervention est plausible dans des conditions réelles d’exercice est une condition incontournable pour sa MAE. L’intervention doit donc permettre une projection opérationnelle aisée dans toute la diversité des situations dans lesquelles son déploiement est envisagé. [Expérimentateurs 1, 2, 3, 4, 5, 6, 8, 9, 10, 11, 13, 14, 15, 16, 17, 21, 22].

#### L’atout «collectif»

Plusieurs mérites sont reconnus à l’inscription de l’intervention dans une démarche collective: addition de compétences variées, création de dynamique de groupe entretenant la motivation autour du projet, répartition des missions et allègement des charges de travail individuelles. Elle permet également de faire face aux *turn over* des agents, d’assurer la continuité du projet et favorise des évolutions de pratiques harmonieuses autour des bénéficiaires. [Expérimentateurs 1, 2, 3, 11, 13, 14, 15, 16, 18, 21].

#### À point nommé

Le potentiel de MAE de l’intervention est renforcé lorsque la problématique traitée correspond à l’agenda politique. Il peut alors s’agir d’opportunités providentielles ou provoquées. Dans le premier cas on observe une contingence programmatique propice. Dans le second cas, sont déployées des stratégies de séduction politique variées. [Expérimentateurs 1, 2, 3, 4, 5, 6, 7, 8, 9, 10, 11, 13, 14, 15, 16, 17, 22].

#### Au gré des interlocuteurs

Les personnes facilitatrices sont des personnes très engagées voire militantes, des personnes fortement et durablement investies pour l’intervention, des personnes influentes exerçant dans les hautes sphères de la société ou ayant un riche réseau de connaissances mobilisables. Enfin, la MAE se fait aussi à la faveur de bonnes relations interpersonnelles. [Expérimentateurs 1, 3, 4, 5, 6, 7, 8, 10, 11, 12, 13, 14, 17, 18, 19, 20].

### Les inhibiteurs de la mise à l’échelle

La MAE peut également se confronter à des conditions défavorables liées à l’intervention, aux ressources disponibles, ainsi qu’aux conditions contextuelles.

#### Des interventions «trop gourmandes»

L’ampleur des moyens humains, temporels et financiers nécessaires est la première difficulté évoquée. Les interventions demandent d’importants efforts de la part des sites d’implantation en se surajoutant à leurs missions basales. Ces investissements sont liés à la formation, à l’appropriation des notions, des outils et des techniques de l’intervention, à l’animation et parfois à son évaluation ou à son suivi. [Expérimentateurs 1, 2, 4, 14, 16, 22].

#### Des mises à l’échelle sous dotées

Le nombre d’agents démultiplicateurs et leur disponibilité plafonnent l’expansion de l’intervention. La méconnaissance et la sous-estimation des activités nécessaires à la MAE engagent des charges de travail importantes et mal anticipées. Les outils nécessaires à l’implantation de l’intervention font parfois défaut. L’invisibilisation de ces activités et la rareté de leur financement entravent encore la MAE. De plus, elles requièrent des compétences rares et insuffisamment valorisées rendant les postes inattrayants. [Expérimentateurs 1, 5, 7, 9, 10, 11, 13, 14, 16, 20, 21].

#### De l’instabilité

Tous les types d’acteurs concernés par la MAE connaissent un fort *turn-over*. Cette inconstance fragilise la MAE de l’intervention. Elle la ralentit également en multipliant les nécessaires périodes de rencontres, de découvertes ou d’appropriations. [Expérimentateurs 1, 4, 5, 7, 10, 17, 18, 22].

#### Une polyphonie dissonante

Il n’y pas d’autorité politique, de chef de cœur dont le rôle serait d’harmoniser les décisions autour de l’intervention et ainsi d’en faciliter la MAE. Le porteur devra prêcher son intervention auprès de multiples interlocuteurs et s’arranger tantôt de son adhésion, tantôt de son refus et ce avec des interlocuteurs d’échelles semblables ou différentes. [Expérimentateurs 1, 2, 3, 6, 7, 10, 11, 18, 19, 20].

#### Une vision court-termiste

Le financement des interventions de santé publique s’inscrit dans une perspective de courte échéance qui va à l’encontre de l’ambition portée par la MAE. Les interventions sont financées via des appels à projets, pour une ou deux années. Dans le meilleur des cas elles s’intègrent dans une convention pluriannuelle d’objectifs et de moyens (CPOM) qui leur accorde une durée de financement de deux ou trois années. Seules les interventions intégrées dans le dispositif article 51 de la LFSS 2018 épousent une perspective de long terme par l’inscription des nouvelles organisations dans le droit commun. Par ailleurs, d’importants déphasages existent entre les calendriers des financeurs et ceux des acteurs. Enfin les financements sont très souvent envisagés comme des amorces supposant que le succès de la MAE relève d’une bonne impulsion, occultant les activités socles nécessaires au processus. [Expérimentateurs 1, 2, 4, 5, 6, 9, 11, 17, 19, 21].

#### Un «marché» concurrentiel

La MAE des interventions de santé publique a lieu dans un paysage qui n’est pas exempt de rivalités. De nombreux expérimentateurs décrivent cette dynamique concurrentielle qui oppose différents éléments. On peut identifier une tension entre différentes thématiques de santé publique. Le financeur, l’opérateur, le site d’accueil peut donc faire le choix de sélectionner la thématique au cœur de l’intervention ou de l’écarter. On peut identifier une tension entre différentes interventions portant sur la même thématique, ciblant la même population. La concurrence entre les différentes interventions existe dans l’attribution des crédits principalement, mais aussi dans le choix de l’acteur démultiplicateur de développer l’une ou l’autre intervention et dans la sélection des sites d’accueil ou des opérateurs. On peut identifier une tension entre différents acteurs démultiplicateurs engagés dans le déploiement d’une même intervention. Ils doivent alors nécessairement s’accorder sur la répartition des zones de dissémination. On peut enfin identifier une tension résultant de contraintes temporelles: entre l’intervention et d’autres activités. Cette rivalité est observée au sein des sites de déploiement, notamment intervention versus missions principales. Elle est également observée auprès des acteurs démultiplicateurs qui sont fréquemment engagés sur plusieurs missions ou interventions. [Expérimentateurs 1, 2, 4, 6, 7, 9, 10, 11, 12, 13, 14, 17, 19, 20, 22].

## Discussion

Cette étude a permis de recueillir les récits de 27 expérimentateurs: concepteurs, opérateurs, chercheurs, évaluateurs ou référents. Au travers de 22 entretiens ce sont 19 processus de MAE qui ont été narrés. La MAE correspond à un processus multidimensionnel, dynamique et graduel. Elle repose sur des stratégies concernant l’expansion territoriale, la pérennisation et l’adéquation au réel. Elle s’appuie sur différentes méthodes permettant d’identifier les démultiplicateurs, ainsi que sur diverses conformations organisationnelles. La MAE est supportée par des décisions favorables émanant de multiples protagonistes qui sont de l’ordre du financement, du soutien, de l’engagement et de l’adoption. Elle se compose d’un ensemble de 8 activités essentielles. La MAE d’une intervention de santé publique suppose des démarches évaluatives autour de plusieurs objets: l’intervention elle-même, son implantation, sa dissémination et sa pérennisation. Elle questionne les méthodes évaluatives employées et particulièrement les dispositifs expérimentaux. Enfin, 6 catalyseurs et 6 inhibiteurs de la MAE sont identifiés. Ils influencent les décisions, les stratégies et les activités.

### Un concept aux multiples réalisations

Les processus de MAE narrés par les expérimentateurs présentent des points communs mais aussi de nombreuses disparités. On observe notamment que la diversité des stratégies de MAE existantes donne naissance à des traductions opérationnelles variées. L’hétérogénéité des situations décrites pourrait menacer la stabilité du concept. Aussi la pluralité de ces expériences invite à distinguer les éléments cardinaux du concept des variations empruntées (tableau 4). Les invariants fondent le concept, tandis que les fluctuations donnent à voir les concrétisations plausibles.

**Tableau 4:** Eléments cardinaux et fluctuations opérationnelles du processus de mise à l'échelle

Mise à l’échelle	
Eléments cardinaux	Fluctuations opérationnelles
Augmentation de la couverture de l’intervention	Echelles géographiques et proportions de populations concernées
Maintien de l’intervention dans le temps	Intégration dans les us et pratiques courantes ou multiples reconductions de financement
Mise en œuvre de l’intervention dans des conditions réelles	Délimitation des éléments fondamentaux de l’intervention
Processus actif	Ressources mobilisées et stratégies arrêtées

### Ordonner la multiplicité des conditions pour la mise à l’échelle

L’analyse des récits a permis de distinguer diverses activités nécessaires à la MAE et plusieurs conditions lui étant favorables. Cependant, elle n’a pas permis de dégager une forme de pondération entre ces éléments. En santé publique comme dans d’autres domaines, la décision exige de prendre en considération de nombreux critères, la décision de MAE d’une intervention n’est pas dispensée de cet impératif (Zoller, 1984). De ce fait, l’intransigeance est écartée, «*les compromis sont inévitables puisqu’il est rarement possible de dégager une solution qui soit optimale par rapport à chacun des critères»,* ainsi on recherchera la ou les solution(s) acceptables à défaut de pouvoir sélectionner la solution optimale (Zoller, 1984)*. *Les plus cartésiens se tourneront vers des méthodes d’analyse multicritère, définies comme *«un ensemble de règles systématiques permettant, à partir d’un certain nombre de critères prédéfinis, de générer des éléments d’aide à la décision. Ces règles décrivent comment les informations fournies par chaque critère sont associées pour pouvoir comparer les objets de l’analyse»* (Karr et al., 2014)*.* De telles analyses permettent de rationaliser les décisions en en décrivant les logiques au moyen de pondérations critériées, de seuils de vétos, d’indifférence ou de préférence (Karr et al., 2014). D’autres méthodologies d’aide à la décision peuvent être saisies telle que l’approche intégrative ou le surclassement. «*Ces méthodes aboutissent soit à une partition (solutions acceptables et solutions non acceptables), soit à un classement plus ou moins ferme des solutions, depuis les meilleures jusqu’aux moins bonnes.* (Zoller, 1984)*»*

### Agir sur l’agenda politique

La concordance entre l’intervention proposée et l’actualité politique est maintes fois identifiée comme un levier. La sociologie politique portant sur les processus de mise à l’agenda nous permet d’envisager cette synchronie sous de nouveaux angles. «*La mise sur agenda est indissociable d’un processus de délimitation et de hiérarchisation des problèmes, de distribution des responsabilités, de recherches de solutions*» (de Maillard & Kübler, 2016). L’avancement d’une solution viable est une condition de la mise à l’agenda du problème auquel elle est destinée. La définition des problèmes à inscrire à l’agenda politique tient compte de la causalité du problème constaté, de son importance, des populations concernées et des solutions potentiellement mobilisables—disponibilité, réalisme de mise en œuvre, acceptabilité et accessibilité sont scrutés (Rochefort & Cobb Roger, 1996). Il n’existe pas de hiérarchie «naturelle» des problèmes qu’il s’agirait de découvrir ou de révéler, l’agenda politique résulte d’une construction intellectuelle et tactique à laquelle plusieurs protagonistes participent (de Maillard & Kübler, 2016). Les opérateurs de santé publique peuvent agir sur l’agenda. Demeurent encore les difficultés liés aux multiples agendas politiques existants concomitamment: locaux, régionaux, nationaux ou supranationaux, qui se recoupent parfois mais sont loin de se confondre (de Maillard & Kübler, 2016).

### Tabou économique

Si la MAE repose sur un impératif économique, les décisions en faveur de la MAE d’une intervention n’exigent nullement de considérations médico-économiques. L’étude met en évidence un paradoxe centré sur les aspects économiques de la santé publique: incontournables sur le principe, laissés pour compte dans la pratique. En effet les aspects médico-économiques c’est-à-dire liés au coût-efficacité des interventions disparaissent des questions évaluatives pour la MAE pour la plupart des interventions étudiées à l’exception des expérimentations intégrées au dispositif article 51 de la LFSS pour 2018 qui impose d’explorer cette dimension et de quelques autres qui ont à cœur de sonder la question et sont en quête de méthodes appropriées. A la condition d’efficience de l’intervention pour la MAE, s’ajoute la question du coût de la MAE elle-même. Celle-ci est rarement explorée—à l’exception encore des interventions développées dans le dispositif article 51—alors qu’elle est considérée comme le premier facteur limitant de la MAE.

### Du renouveau au service de l’adoption et de la longévité

En l’absence de décision astreignante, la MAE repose sur la volonté des opérateurs et donc sur leur séduction ou leur conviction. La «nouveauté» de l’intervention est un argument favorable fréquemment cité. Les sciences de gestion et de marketing ont investi cette thématique sous la dénomination de «comportements exploratoires» (Langlet & Giannelloni, 2004). Parmi les recherches menées sur le sujet, Venkatraman et McInnis distinguent les comportements exploratoires épistémiques des comportements exploratoires sensoriels (Venkatraman & MacInnis, 1986). Les premiers sont liés à une appétence de savoirs. Les seconds sont orientés vers la quête de sensations. L’adoption et la mise en œuvre d’une intervention de santé publique peuvent découler d’un attrait sensoriel (via de nouvelles postures, techniques ou outils d’animation par exemple) ou cognitif (concernant le contexte épidémiologique par exemple ou la théorie de l’intervention sous-jacente), aussi il nous semble intéressant d’investir davantage ces travaux.

### Un décideur dispersé

La MAE d’une intervention de santé publique ne découle pas du choix d’un unique protagoniste qui serait maitre du processus. Ce constat n’est ni propre au processus de MAE ni au domaine de la santé publique. Il concerne plus globalement l’action publique qui est définit comme une construction collective d’acteurs en interaction (Hassenteufel, 2011). L’analyse de l’action publique invite à interroger la notion même de «décideur». La diffraction de la décision s’ajoute à son morcellement: «*le vocable «décideur» recouvre en réalité des intervenants dont les intérêts et priorités ne sont pas forcément convergents»* (Zoller, 1984)*. *Pour certain, «*l’histoire de la discipline* (l’analyse de l’action publique) *peut être lue comme une déconstruction progressive du mythe du décideur rationnel, omniscient et omnipotent»* (Belorgey, 2012)*.* Plusieurs éléments contribuent à la dispersion de la décision dans le champ de la santé publique. Tout d’abord la complexité du système de santé français qui s’explique par son historique de construction «*par vagues successives*» et «*sans véritable architecte*» et qui se traduit par une multitude d’organes de pilotage enchevêtrés (Tabuteau, 2008). Par ailleurs les acteurs influençant la santé de la population sont présents dans divers secteurs d’activité (Menvielle & Lang, 2021). Ce constat nous invite à mener une analyse des processus de MAE des interventions de santé publique selon les canons de la sociologie politique de l’action publique en passant par l’identification des acteurs en présence, l’analyse de leurs interactions et leur contextualisation (Hassenteufel, 2011). La littérature sur la MAE voit également apparaitre cette invitation (Larouche et al., 2022). Nous pourrions également étudier l’adoption de méthodes de consensus dans le cadre de la MAE d’intervention de santé publique. Ces méthodes permettent de considérer un large éventail de positions, de les organiser et de les structurer afin de prendre des décisions. *«Le consensus, comme méthode de production de connaissances ou de prise de décision, met l’accent sur l’importance de l’opinion de chaque participant et permet d’exprimer un résultat qui ne fait pas l’objet d’une opposition formelle.»* (Bourrée et al., 2008)*.* Les conférences de citoyens ou les PubliForum par exemple, variantes de la conférence de consensus, semblent des pistes prometteuses, permettant de marier les différents points de vue (Bourrée et al., 2008).

### Le chant de l’hydre

Certains acteurs parviennent brillamment à composer avec la polyphonie dissonante à laquelle la MAE fait face. Leurs profils pourraient correspondre à ce que certains sociologues ont dépeint sous le titre d’*entrepreneur-frontière* (Bergeron et al., 2013). Le concept d’entrepreneur caractérise l’acteur qui tente de problématiser et porter un problème sur la scène publique. En proposant de mettre à l’échelle une nouvelle intervention pour la santé des populations; le concepteur, porteur ou porte-parole à titre individuel, ou la structure support, à tire collectif, endosse un rôle d’entrepreneur. La figure de l’entrepreneur a fait l’objet de multiples travaux sociologiques. Bergeron, Castel et Nouguez propose d’en retenir trois profils (Bergeron et al., 2013). L’entrepreneur «passeur» et l’entrepreneur «traducteur» présentent une capacité remarquable à s’affranchir ou à transcender les frontières séparant les différents milieux concernés par l’entreprise (ici l’entreprise correspond à l’intervention et à sa MAE) (Bergeron et al., 2013)*.* Contrairement aux deux autres, l’entrepreneur frontière ne cherche pas à amoindrir les frontières, il les sublime car elles sont le terreau fertile de son entreprise (Bergeron et al., 2013). Il cultive le morcellement, en en faisant sa stratégie d’évitement de conflits. Il nourrit de multiples identités qui sont autant d’alliés pour conquérir ses interlocuteurs. Chaque facette de la personnalité, de l’histoire ou de la formation de l’agent pourra être investie pour gagner son audience. Au sein d’une structure collective, chaque collaborateur sera utilement exploité dans le milieu qui est le sien. L’entrepreneur «frontière» n’hésite pas à multiplier les partitions plutôt qu’à rechercher la mélodie qui séduirait toutes les oreilles. A la discrétion de l’interlocuteur de retenir ou d’omettre l’une ou l’autre tête de l’hydre entrepreneur. L’analyse sociologique des principaux acteurs ou structures en cause dans le développement de l’intervention ainsi que leur profilage à l’aune des concepts d’entrepreneurs et d’entreprenariats permettraient d’identifier les caractères gagnants capables d’assurer le processus de MAE.

### Entre science des solutions et science du solutionnement

L’intervention de santé publique (programme et politique) est l’objet d’étude de la recherche interventionnelle pour la santé des populations (RISP) (Potvin et al., 2013). C’est donc à la RISP que sont adressées les préoccupations centrées sur la MAE des interventions. Que peut-on attendre précisément de la RISP face à cette finalité? En 2013, dans un article qui fait aujourd’hui référence dans le domaine, Potvin et al. consacraient le terme de «science des solutions» à la RISP (Potvin et al., 2013). En la nommant ainsi, Potvin et al. ne bousculaient pas seulement «le mythe tenace et pernicieux» selon lequel l’acquisition de savoirs sur les problèmes de santé publique suffirait à la décision et à l’action. Si cette seule bascule était ciblée, les auteurs auraient pu baptiser la RISP «science des interventions». Il n’en est rien. En choisissant l’expression «science des solutions», Potvin et al. ont proposé deux translations. La première consistait à déplacer la focale positionnée sur les problèmes de santé publique vers les interventions pour la santé publique. La seconde translation proposait de migrer des connaissances portant sur les interventions aux connaissances abreuvant les solutions. Autrement dit, la RISP propose de concourir à la résolution ou à l’amoindrissement des problèmes de santé publique, non pas en présentant des interventions mais des solutions, elles-mêmes nourries par les savoirs acquis des interventions étudiées. Aussi le terme «science du solutionnement» aurait pu être proposé afin de réduire la tentation d’attendre des interventions qu’elles soient elles-mêmes les solutions. C’est bien cela, nous semble-t-il, qui était proposé par Potvin et ses collègues via les énoncés suivants: «*la RISP met l’accent sur le développement d’un corpus de connaissances sur des interventions ou des stratégies pour intervenir sur une question*», «*Ce qui nous manque encore cruellement c’est une gamme de moyens éprouvés[…] Investir dans la RISP c’est se doter de moyens scientifiques pour réfléchir et orienter les actions nécessaires pour promouvoir la santé des populations*». La ou les solution(s) sont ici envisagées comme des recréations, des recompositions à partir des savoirs issus des interventions expérimentées ou expertisées.

D’un autre côté, pour le medical research council, la recherche sur les interventions complexes a pour but de fournir des interventions «applicables, rentables, transférables et généralisables dans des conditions réelles» (Skivington et al., 2021). Ainsi, on peut supposer que le MRC quant à lui, entend la RISP comme une science des interventions.

**Fig. 3:**
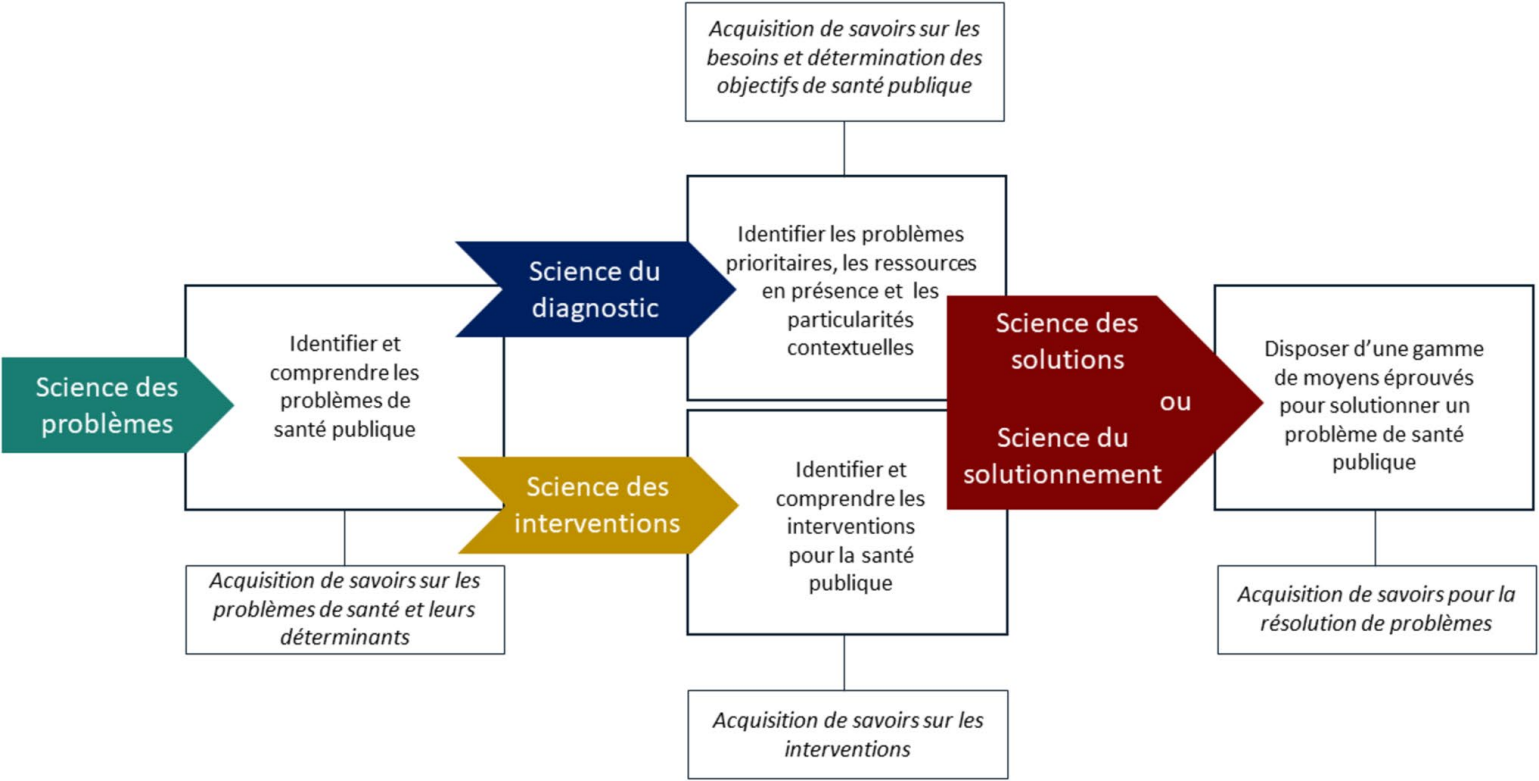
Science des problèmes, science du diagnostic, science des interventions et science des solutions ou du solutionnement

La façon dont on considère la RISP—comme étant une science des solutions ou une science des interventions—a des implications majeures sur la manière dont on adressera les questions de MAE. On retrouve là l’affrontement entre le «sur mesure» et le «prêt à porter». La MAE d’interventions ou la généralisation de solutions. Cela rejoint également les questionnements autour de l’approche linéaire orientée intervention et l’approche systémique orientée système proposé récemment dans la littérature sur la MAE (Koorts & Rutter, 2021). La MAE des interventions est confrontée à de nombreux obstacles, la littérature en identifie généreusement de même que notre recherche (Zomahoun et al., 2019). Parmi les difficultés rencontrées, celles de la viabilité de l’intervention ou du réalisme de son application, et celles de son adaptation aux différents contextes sont prégnantes. L’une des voies permettant de braver ces difficultés est peut-être de se rallier plus concrètement à l’approche proposée par Potvin et al. En investissant complètement cette conception de la RISP, nous serions amenés à quitter l’ambition systématique de mettre à l’échelle des interventions. En revanche nous serions sans doute invités à généraliser la prise en charge des problèmes, le diagnostic de santé publique et l’apport de solutions par l’élaboration de stratégies interventionnelles ancrées, comme le suggèrent certains acteurs rencontrés dans cette étude. Le point d’entrée de la solution ne serait plus l’intervention mais le diagnostic du milieu. Le processus irait des objectifs situés vers des activités ancrées. Cette voie présente également l’intérêt de prendre en compte les nombreux aspects concurrentiels qui se dressent face à l’intervention. Selon cette approche, la MAE est reconnue comme un processus complexe qui s’insère et bouscule des activités et des organisations existantes, une réflexion structurée est nécessaire à son entreprise et ne porte pas seulement sur l’intervention ciblée ou sur les aspects techniques du processus de MAE (Larouche et al., 2022). Cela étant dit, la science des interventions demeure incontournable, mais elle devrait être complétée par une science du diagnostic qui permette de discerner les savoirs utiles acquis sur les interventions, toutes deux menant à une véritable science du solutionnement qui permette de répondre aux problèmes de santé publique identifiés dans les territoires en tenant compte des contextes et des priorités établies, dans une incertitude maitrisée (Fig. 3). C’est ainsi redonner tout son sens au processus de MAE lui-même qui est du côté de la MAE de l’impact et non de l’intervention (McLean & Gargani, 2019), les deux ambitions n’étant pas inconciliables évidemment. C’est éviter le piège du «plus égale mieux» au bénéfice d’une pratique de la MAE plus raisonnée (McLean & Gargani, 2019).

### Forces et limites

Pour la majorité des acteurs rencontrés, leurs emplois étaient totalement ou partiellement conditionnés par l’intervention narrée et sa MAE. Seules 3 des 27 interviewés n’étaient plus impliquées dans les interventions au cœur des récits lors du recueil d’expériences.

Notre analyse ne pointe pas avec précision les profils des expérimentateurs rencontrés. Deux raisons à cela: l’anonymisation des récits à la faveur d’une libre expression, ainsi que la difficulté à cerner des parangons à partir des données recueillies. En effet nos interlocuteurs arboraient des fonctions peu discriminantes: par le titre (chargé de projet/de mission, chef de projet, référent, etc.) et le cumul ou la succession (missions différentes autour de l’intervention, au sein d’une ou de différentes structures).

Nous avons considéré des interventions et MAE variées en divers points. En revanche nous avons étudié uniquement des expériences françaises. La part des variables susceptibles d’influencer la MAE relative au contexte national a ainsi été neutralisée (plans sanitaires, économiques, législatifs et politiques).

### Perspectives

Cette étude nous invite à envisager de nouvelles perspectives de recherche. Il serait intéressant d’explorer les pratiques des institutions et des financeurs publics, les dispositifs dans lesquelles elles s’expriment et les contraintes qui les encadrent. Nous pourrions également explorer les phases de délibération concernant la MAE. Une analyse différentielle des processus de MAE d’interventions d’approche populationnelle d’une part et des interventions d’approche individuelle d’autre part pourraient être réalisée, de même en et hors milieu de soins ou en et hors milieu scolaire. D’autres caractéristiques propres à l’intervention pourraient également être examinées plus avant; notamment la granularité systémique de l’intervention ainsi que sa valence prescriptive et leurs incidences sur le processus de MAE tant ces caractéristiques influent sur le fragile équilibre entre respect de l’intégrité et adaptation de l’intervention, nœud majeur de la MAE. Par ailleurs, cette étude montre une cristallisation autour de la qualification d’intervention «probante» qui apparait comme un sésame de la MAE. Cependant les conditions de cette appellation ne sont ni évidentes ni consensuels. Ainsi un nouveau pan de cette recherche pourrait se centrer sur les attentes, représentations et pratiques de différentes parties prenantes de la MAE à ce sujet. Enfin, nos recherches pourraient investir une échelle internationale: par l’étude des interventions MAE sur un plan international ou par la comparaison entre différentes nations.

## Conclusion

Résolument tournées vers l’utilisation de données probantes, les politiques publiques de santé publique ont tout à miser sur la MAE des interventions. Cependant, si cette ambition est assez largement partagée, son opérationnalisation n’est pas un acquis. Les parties prenantes de la MAE nécessitent d’être mieux informées sur le processus, ses variantes et ses impératifs. La littérature propose un certain nombre de modèles, de guides et d’outils riches d’informations et de conseils pour la MAE des interventions de santé publique. Force est de constater qu’ils ne permettent cependant pas de surmonter les difficultés qui entravent le processus de MAE. C’est pourquoi nous nous sommes tournés vers les expérimentateurs de la MAE en contexte français afin de décrire finement ce qu’il se produit dans le monde réel, afin de capter les dynamiques, difficultés et facilités perçues par les acteurs. Des entretiens semi-directifs ont été réalisés auprès de 27 expérimentateurs de la MAE endossant actuellement ou par le passé diverses casquettes dans le processus. La MAE correspond à un processus actif, progressif, qui se déploie dans les trois dimensions suivantes: l’espace, le temps et le réel. Elle repose sur différents types de stratégies: certaines concernent l’expansion territoriale, d’autres la pérennisation de l’intervention et d’autres encore l’adéquation au réel. Huit activités, constitutives de la MAE, sont déployées tout au long du processus. De plus la MAE s’appuie sur une organisation qui peut épouser différentes conformations et différentes méthodes d’identification des agents démultiplicateurs. Plusieurs types de décideurs et plusieurs formes de décisions influencent le processus: financement, soutien, engagement et adhésion favorisent la MAE. Enfin les récits des expérimentateurs ont permis de souligner 6 catalyseurs et 6 inhibiteurs particulièrement influents sur le processus de MAE. Les résultats de cette étude confirment, complètent et précisent les propositions fournies par la littérature. Concernant le concept de MAE, les résultats de notre étude soulignent une nouvelle fois le caractère actif de la MAE, et son expression dans les trois dimensions de l’espace, du temps et du réel. Ils précisent les frontières concernées effectivement par la MAE et les motifs de ces délimitations. Ils appuient sur le caractère continu de la MAE tant que le besoin perdure et insistent sur sa nature progressive. Concernant le processus de MAE, notre étude confirme son aspect non linéaire et offre des précisions sur les différentes stratégies adoptées et adoptables, sur les décisions soutenant la MAE, sur l’organisation de ce processus ainsi que sur les activités sous tendues par la MAE, et enfin sur plusieurs objets évaluatifs. Concernant les freins et les leviers identifiées, s’ils ne contredisent pas ceux identifiés dans la littérature, ils permettent de développer certains aspects et de les rendre plus tangibles et ancrés dans le contexte français actuel.

## Contributions a la connaissance

Qu’est-ce que cette étude ajoute aux connaissances existantes?

- Ces travaux précisent le concept même de mise à l’échelle des interventions complexes en santé publique
- Ils décryptent la nature et les arcanes du processus de mise à l’échelle notamment sur les aspects de stratégies, de décisions et d’activités
- Ils renforcent et complètent les connaissances existantes sur les freins et leviers à la mise à l’échelle des interventions de santé publique

Quelles sont les principales conséquences qu’il y a lieu d’en tirer pour les interventions, la pratique ou les politiques en santé publique?

- Ces travaux guident les différents acteurs engagés dans le processus de mise à l’échelle d’une intervention. En effet, non seulement ils soulignent résolument le caractère actif du processus de mise à l’échelle mais proposent également de le décortiquer en stratégies, en organisations et en activités.
- En s’intéressant tant au concept qu’au processus de mise à l’échelle des interventions de santé publique, instruments importants de l’action publique dans le domaine, ces travaux contribuent à améliorer le design et la mise en œuvre des politiques de santé publique fondées sur les données probantes.

## Remerciements

Ce manuscrit est issu des travaux de thèse du premier auteur, soutenue le 4 novembre 2024.

Nos remerciements à l’ensemble des acteurs qui nous ont fait part de leurs expériences.

## Data Availability

Les détails de l’analyse thématique peuvent être fournis par l’auteur correspondant sur demande.

## Footnotes

1 En France, il s’agit d’une mission confiée par le ministre de la santé Olivier Véran au professeur Franck Chauvin ayant pour objectif de «Refonder» la santé publique. https://sante.gouv.fr/ministere/acteurs/instances-rattachees/mission-refondation-de-la-sante-publique-dessiner-la-sante-publique-de-demain/

2 En France, la loi de financement de la sécurité sociale pour 2018 a introduit, en son article 51 un dispositif permettant l’expérimentation de nouvelles organisations en santé dans une visée d’intégration dans le droit commun. https://sante.gouv.fr/systeme-de-sante/parcours-des-patients-et-des-usagers/article-51-lfss-2018-innovations-organisationnelles-pour-la-transformation-du/article-51

3 L’agence Santé Publique France propose un portail d’interventions efficaces ou prometteuses en prévention et promotion de la santé, incrémenté progressivement suite à des évaluations conduites par un comité d’experts. https://www.santepubliquefrance.fr/a-propos/services/interventions-efficaces-ou-prometteuses-en-prevention-et-promotion-de-la-sante

4 En France, la Société Française de santé publique pilote une démarche collective visant à proposer un espace d’échanges autour du déploiement des interventions. https://www.sfsp.fr/content-page/item/63836-dipp-deploiement-des-interventions-probantes-et-prometteuses-en-prevention-et-promotion-de-la-sante

## Disponibilité du code

Sans objet.

## Declarations

### Approbation éthique

Sans objet.

### Consentement à la participation

Le consentement éclairé de tous les participants a été obtenu.

### Consentement à la publication

Sans objet.

### Conflit d’intérêts

Aucun à déclarer.
